# Role of Genetic Variation in ABC Transporters in Breast Cancer Prognosis and Therapy Response

**DOI:** 10.3390/ijms21249556

**Published:** 2020-12-15

**Authors:** Viktor Hlaváč, Radka Václavíková, Veronika Brynychová, Renata Koževnikovová, Katerina Kopečková, David Vrána, Jiří Gatěk, Pavel Souček

**Affiliations:** 1 Toxicogenomics Unit, National Institute of Public Health, 100 42 Prague, Czech Republic; viktor.hlavac@szu.cz (V.H.); rvaclavikova@seznam.cz (R.V.); veronikabrynychova@seznam.cz (V.B.); 2 Biomedical Center, Faculty of Medicine in Pilsen, Charles University, 323 00 Pilsen, Czech Republic; 3 Department of Oncosurgery, Medicon Services, 140 00 Prague, Czech Republic; renata.kozevnikovova@onko-centrum.cz; 4 Department of Oncology, Second Faculty of Medicine, Charles University and Motol University Hospital, 150 06 Prague, Czech Republic; katerina.kopeckova@fnmotol.cz; 5 Department of Oncology, Medical School and Teaching Hospital, Palacky University, 779 00 Olomouc, Czech Republic; davvrana@gmail.com; 6 Department of Surgery, EUC Hospital and University of Tomas Bata in Zlin, 760 01 Zlin, Czech Republic; gatekj@gmail.com

**Keywords:** ABC transporter, therapy response, disease-free survival, breast cancer, next-generation sequencing, competitive allele-specific PCR

## Abstract

Breast cancer is the most common cancer in women in the world. The role of germline genetic variability in ATP-binding cassette (ABC) transporters in cancer chemoresistance and prognosis still needs to be elucidated. We used next-generation sequencing to assess associations of germline variants in coding and regulatory sequences of all human ABC genes with response of the patients to the neoadjuvant cytotoxic chemotherapy and disease-free survival (*n* = 105). A total of 43 prioritized variants associating with response or survival in the above testing phase were then analyzed by allelic discrimination in the large validation set (*n* = 802). Variants in *ABCA4, ABCA9, ABCA12, ABCB5, ABCC5, ABCC8, ABCC11,* and *ABCD4* associated with response and variants in *ABCA7, ABCA13, ABCC4*, and *ABCG8* with survival of the patients. No association passed a false discovery rate test, however, the rs17822931 (Gly180Arg) in *ABCC11,* associating with response, and the synonymous rs17548783 in *ABCA13* (survival) have a strong support in the literature and are, thus, interesting for further research. Although replicated associations have not reached robust statistical significance, the role of ABC transporters in breast cancer should not be ruled out. Future research and careful validation of findings will be essential for assessment of genetic variation which was not in the focus of this study, e.g., non-coding sequences, copy numbers, and structural variations together with somatic mutations.

## 1. Introduction

Breast cancer (Online Mendelian Inheritance in Man, OMIM no. 114480) is the most common cancer in women in the world [1]. One of the frequently studied reasons for the lack of successful treatment outcomes in a considerable portion of the patients is multidrug resistance [2]. Multidrug resistance can be caused by ATP-binding cassette (ABC) transporters, e.g., by higher efflux of drugs out of tumor cells by P-glycoprotein (Multidrug resistance, MDR coded by *ABCB1* gene) [2,3].

ABC transporters represent a large superfamily of membrane transporter proteins classified into seven families and translocate numerous compounds across intra and extracellular membranes. Their substrates include metabolic products, sterols, lipids, and xenobiotics [3]. However, of the total number of 48 active human ABC transporters, up to only 16 are able to transport anticancer drugs [3]. Most of the drug resistance is ascribed to largely studied multidrug resistance-related transporters *ABCB1* (MDR, OMIM no. 171050), *ABCC1* (MRP, OMIM no. 158343), and *ABCG2* (BCRP, OMIM no. 603756) [4]. Proteins of the family ABCA are mostly lipid sterol transporters and can be associated with several diseases, e.g., Tangier or Alzheimer disease [5]. Their roles in cancer progression and the metastatic potential linked to lipid trafficking recently became the focus of numerous studies [6]. ABCBs participate in antigen processing and immune deficiency. Apart from ABCB1, a promiscuous and ubiquitous efflux pump [2], ABCB members also represent transporters of heme and bile acids [7]. Family C is mostly dedicated to multidrug resistance (MRP1-6) [8,9], but ABCC6, ABCC7, and ABCC8/9 are linked to serious diseases (pseudoxanthoma elasticum, cystic fibrosis, and diabetes mellitus, respectively) [9]. ABCDs are responsible for transport of fatty acids from peroxisomes to the cytoplasm [10] and ABCGs transport various products of metabolism, xenobiotics including anti-cancer drugs, bile acids, and steroids [11]. The rest of the transporters are not involved in transport, but rather act as translational inhibitors or protein synthesis regulators (ABCFs and ABCEs) [12].

Our recent pharmacogenomics study revealed a prognostic and predictive potential in a number of alterations in breast cancer [13]. The studied genes were implicated in the metabolism and transport of drugs administered to breast cancer patients in the clinics, clearly documenting the importance of this tool for the personalized medicine. The study provided a proof-of-the principle for the design and bioinformatics methodology, namely, the assembly and testing of an in-house pipeline for variant prioritization. Given the total number of 509 genes screened by the next generation sequencing (NGS), only a portion of variants could be validated in a subsequent phase. In order to select the most relevant functional alterations from the statistically significant set of variants, we down-sampled the results using information from in silico predictions and according to previously confirmed pharmacogenomic and clinical evidence. Thus, some potentially useful biomarkers of prognosis or prediction of therapy outcome could have been missed.

In the present study, we aimed to use less strict criteria for investigating the importance of germline genetic variability in coding, untranslated regions (UTR), and adjacent regions of all human members of the ABC superfamily for prognosis and response to cytotoxic therapy of breast cancer patients. All variants in ABCs identified in the testing phase were correlated with disease-free survival (DFS) and response of the patients to preoperative cytotoxic therapy, and thoroughly reviewed, including permutation analysis, evaluation of haplotypes, and gene dosage. We have not addressed functional relevance to enable identification of purely correlative biomarkers. Prioritized variants were further validated in a large cohort of breast cancer patients from a single population. Thus, the present study brings a more detailed view of the relevance of genetic variability of ABC transporters for breast cancer prognosis and therapy outcome predictions.

## 2. Results

### 2.1. Testing Phase

The clinical characteristics of the patients (*n* = 105) are in Table S1. The subgroup of patients was treated with the neoadjuvant cytotoxic therapy (NACT) (*n* = 68) and the response to this treatment was available. The rest of patients were treated with adjuvant cytotoxic therapy based on monotherapy or combinations of anthracyclines, cyclophosphamide, 5-fluorouracil, and taxanes. DFS was evaluated for all patients and the mean follow-up of the patients was 70 ± 28 months.

The average coverage was 82.3 ± 29.1 and 95% of the captured regions were covered at least ten times. Altogether, we found 2611 variants in exonic and adjacent intronic sequences. Of the total number of 48 genes and one pseudogene (*ABCC13*) subjected to analysis, 46 genes (94%) contained at least one genetic alteration. No alterations were found in *ABCF1*, *TAP1* (alias *ABCB2*), and *TAP2* (*ABCB3*) genes. On the other hand, the most polymorphic genes, with over one hundred alterations, were *ABCA13* (165 alterations), *ABCA4* (114), and *ABCA1* (109). Of the total number of 2611 variants, 636 were in exons, 1544 intronic, and 253 were in 3’UTR or 5’UTR regions according to National Center for Biotechnology Information (NCBI) Reference Sequence Database (RefSeq; https://www.ncbi.nlm.nih.gov/refseq/) in Annovar (Table 1).

On average, each patient showed 608 ± 33 variants. We found 17 loss-of-function (LOF) truncating variants that were either affecting the stop codon (stop-gain) or frameshift insertions or deletions (indels). There were 355 of the variants that were non-synonymous single nucleotide variants (SNVs) and 263 that were synonymous SNVs (Table 2). In total, 1058 variants (41%) had minor allele frequency (MAF) > 0.05, and the rest of the 1553 variants, had MAF ≤ 0.05.

**Figure 1:**
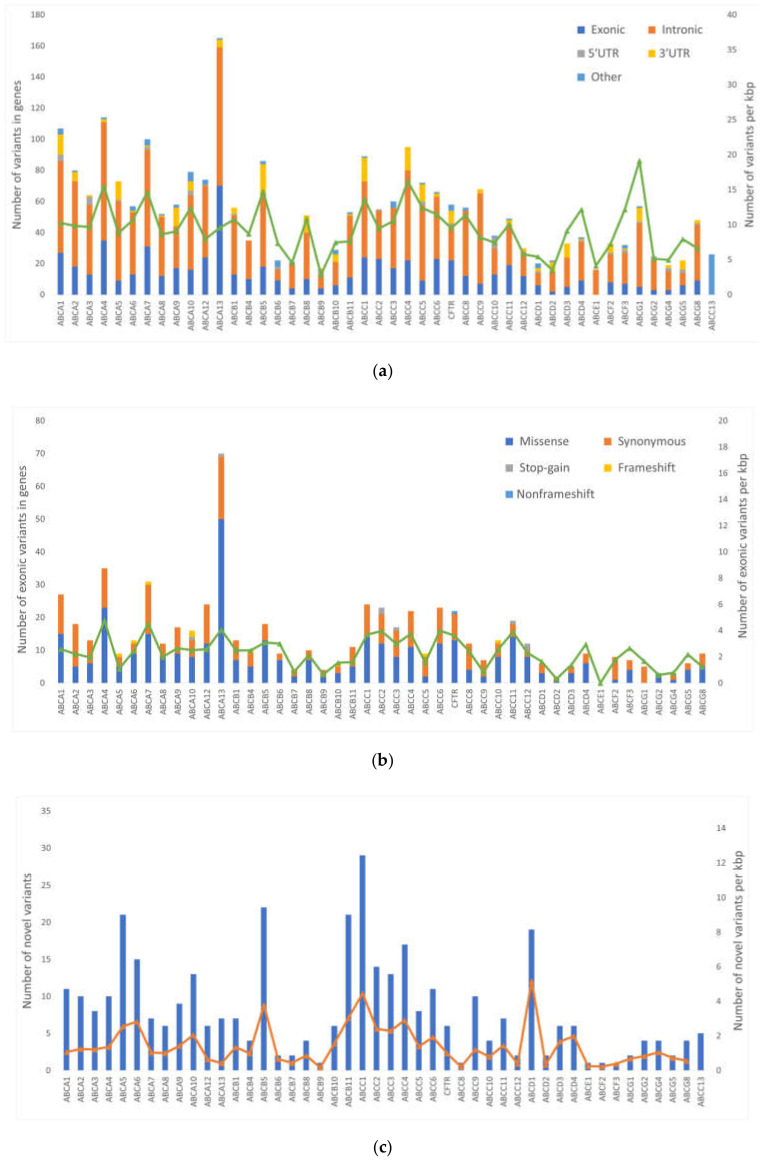
Distribution of alterations in individual ABC transporter genes. The picture shows: (**a**) the frequency of genetic alterations according to their functional classes; (**b**) the frequency of genetic alterations according to their exonic functional classes analyzed by RefSeq: National Center for Biotechnology Information (NCBI) Reference Sequence Database (https://www.ncbi.nlm.nih.gov/refseq/) shown according to individual transporters (excluding *ABCC13* pseudogene); and (**c**) the number of novel variants according to individual transporters. The number of the variants normalized to the transcript length in kilo base pairs (kbp) per each gene are shown for each plot on the right axis and depicted by the overlaid line.

Altogether, 256 (10%) of the variants were novel (i.e., not found in dbSNP Build 150). Out of these, 162 had MAF > 0.01. The distribution of the variants according to their functional classes and frequencies of novel variants in the groups of genes are shown in Figure 1.

Variants departing from Hardy–Weinberg equilibrium (*p* < 0.01, *n* = 101) were excluded from analyses and further only variants with MAF > 0.05 were considered relevant to achieve adequate statistical power in the validation phase. In addition, variants with the missing data in more than 50% of patients were excluded (*n* = 54). Application of these filtration criteria resulted in a set of 903 variants which were further evaluated for associations with the response of patients to NACT and DFS. We found 56 variants associated with the response to NACT and 47 variants associating with DFS. Six variants reported significant in the previous study [13] were further excluded. Following haplotype evaluations, 38 variants were considered tagged (>80%) with some other variant and were not assessed further. As a result, 22 variants associated with the response and 37 variants associated with DFS were followed. The gene dosage relationship was then evaluated for variants associating with DFS and variants not fulfilling this condition were excluded (*n* = 7). Neither of these variants was significant in the recessive genetic model (variant allele versus wild type). Following these control steps, 52 variants (45 SNVs and 7 indels, Table S2) were prioritized for the validation phase in a larger cohort of breast cancer patients.

### 2.2. Validation Phase

The clinical characteristics of the patients (*n* = 802) are summarized in Table S3. A subgroup of patients treated with NACT composed of 168 patients. In total, 371 patients received adjuvant cytotoxic therapy. Patients with localized disease and good prognosis were untreated (*n* = 83) and a portion of patients was treated only with hormonal therapy (*n* = 311). The mean follow-up of the patients was 76 ± 30 months.

Out of 52 variants subjected to genotyping, attempts to optimize detection techniques failed in 10 variants (5 indels and 5 SNVs) and could not be further evaluated for clinical associations. One variant was additionally included into the list (rs2893007) based on haplotype tagging (*r*^2^ = 1) to replace the variant rs11764054 whose analysis failed. No tagging variants (*r*^2^ > 0.8) were found to replace the rest of the failed variants. In the end, 43 variants were successfully genotyped. No variants significantly departed from Hardy–Weinberg equilibrium and less than 1% of theoretical data points were missing due to uncertainty in genotype calling, or an absent signal. MAFs of variants in the validation phase did not substantially differ from those observed in the testing one (Table 3).

We further evaluated associations of variants with the response and DFS of patients in the validation set. For six variants with less frequent homozygous genotypes (*n* < 5), the recessive genetic model was used for the sake of the statistical power of comparisons. The variants that associated with response in both testing and validation phases are listed in Table 4 and thus these variants are considered replicated with putative clinical importance. However, none of these associations passed the false discovery rate (FDR) test for multiple testing (*q* = 0.0025) and, thus, cannot be deemed statistically significant after such correction.

**Figure 2:**
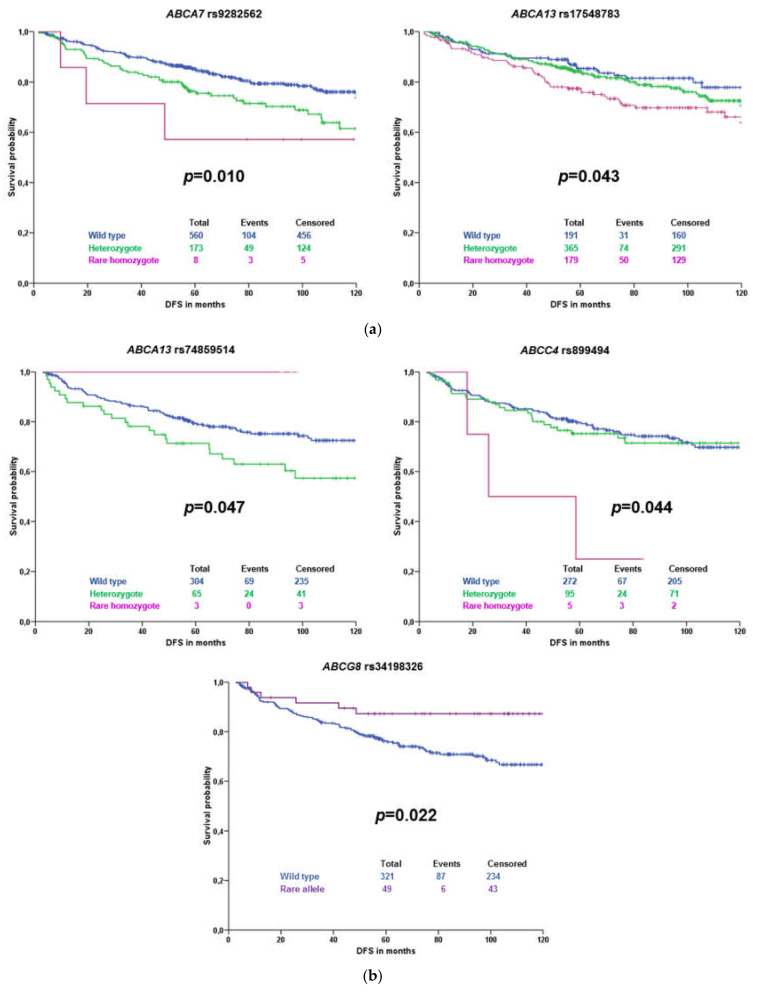
Kaplan–Meier plots with validated associations of variants with disease-free survival (DFS) of all patients unstratified (**a**) or subgroup of patients treated with cytotoxic therapy (**b**). The blue line represents the common homozygous genotype, the green line the heterozygote, and the magenta line the rare homozygote. The violet color represents rare variant carriers. Significance was evaluated by the log-rank test; numbers represent individuals in compared groups.

Subsequently, associations of variants with DFS of all patients and patients stratified according to the received therapy were evaluated. Significant results for all patients with complete follow-up data (*n* = 744) are displayed in Figure 2a. Results for patients treated with cytotoxic therapy (*n* = 371) are presented in Figure 2b. No significant association was observed in a subgroup of patients treated only with hormonal therapy (*n* = 312).

Of these variants, rs74859514 did not pass the gene dosage condition (Figure 2b). None of the associations has passed the FDR test for multiple testing (*q* = 0.0023) and, thus, cannot be further considered statistically validated.

We further clarified the effect of molecular subtype on prognosis of the patients by their stratification according to the intrinsic molecular subtype. Associations with DFS were then calculated separately for each subtype (Table 5 and Figure S1).

This analysis showed that prognostic associations differ according to the molecular subtype. In the whole group of patients, rs9282562 in *ABCA7* and rs17548783 in *ABCA13* were prognostic only for HER2 enriched and triple negative (TNBC) subtypes, respectively. In the subgroup treated with cytotoxic therapy, again single nucleotide polymorphisms (SNPs) were prognostic for patients with HER2 enriched and TNBC subtypes (rs74859514 in *ABCA13*). Carriers of the rare variant in *ABCG8* rs34198326 had better DFS than those with the common homozygous genotype—this association was significant only in patients with the luminal B subtype.

In order to clarify associations of variants with gene expression, we used transcriptomic data from previous gene expression profiling [14] and compared it with variants that significantly associated with DFS or response to NACT in the validation set (*n* = 168 patients for whom gene expression was available). We also analyzed expression quantitative trait loci (eQTL) associations of these variants using gene expression in healthy tissues available on the GTEx portal (https://www.gtexportal.org). The SNP rs17548783 was significantly associated with *ABCA13* transcript levels in tumors assessed in the previous study [14] (Table 6), but no eQTL were found in the GTEx database. No eQTL were found also for rs2275032 in *ABCA4*, rs71428357 in *ABCA12*, rs3210441 in *ABCB5*, and rs34198326 in *ABCG8*. Significant results from eQTL analysis are summarized in Table 7 and Figure S2.

## 3. Discussion

The role of germline genetic variability among ABC transporters in prognosis of breast cancer patients as well as in their response to chemotherapy is underexplored. In our previous publication, we dealt with pharmacologically relevant germline genetic polymorphisms in 509 breast cancer-related genes [13]. In the present study, we used the same approach to reveal all associations of genetic variants in human ABC transporters with chemotherapy response and survival of the patients.

A total of 2611 variants were found in a testing set. The majority of variants were found in intronic regions. Lower numbers of variants were found in coding regions and UTRs. Interestingly, no variants were found in *ABCF1*, *TAP1* (alias *ABCB2*), and *TAP2* (*ABCB3*). *TAP1* and *TAP2* are antigen presenting transporters and alterations in these genes associate with autoimmune diseases, susceptibility to infections, or malignancies [15]. Similarly, *ABCF1* plays a role in the regulation of inflammatory processes [16] and alterations in *ABCF1* are linked with autoimmune diseases as well [17]. Therefore, it seems that genetic variants in these genes negatively impacts immunity and inflammatory processes which explain limited variability, in line with our findings. On the other hand, the most variable genes were *ABCA13* (165 alterations), *ABCA4* (114), and *ABCA1* (109). The members of ABCA family are typically large genes (transcript length 7‒17 kbp) and thus likely to accumulate variants. When normalized for the length of transcript, *ABCG1, ABCC4*, and *ABCA4* have the highest count of variants per kbp, ranging from 16 to 20. Interestingly, *ABCA4, ABCA7*, and *ABCA13* had the highest variant counts in exonic regions (4.1‒4.8 variants per kbp). We found several LOF variants in ABC transporters—eight stop-gains and nine frameshift indels. These events have high impact on function of the protein. Moreover, all 17 LOF variants were present in genes of the first quartile of the most intolerant genes to LOF events [18]. These facts advocate for further investigation of LOF variants in ABC transporters. Unfortunately, LOF variants are rare. For the sake of maintaining enough statistical power for comparison with clinical data, only common variants (MAF > 0.05) could be used in the present study.

In total, we selected 903 variants and subjected them to a thorough statistical analyses. Of these variants, 43 associated with response or DFS and were capable of validation in a cohort of 802 breast cancer patients. Five associations with DFS and nine with response to NACT were replicated in the validation set. If multiple simultaneous statistical tests are calculated, a type I error (a risk of false positive results) occurs. To prevent this error, correction for multiple testing must be used. There are several methods to do so. Here, we applied the wildly used FDR, a test by Benjamini–Hochberg. After this correction, none of the associations of variants with clinical features remained significant and, thus, cannot be considered validated. Nevertheless, we found some interesting associations which we will discuss further.

*ABCA13* is responsible for lipid transport and variants in this gene can cause schizophrenia [5]. Carriage of the rare allele of SNP rs17548783, located in downstream intronic region of *ABCA13,* was associated with shorter DFS of patients in our study. Based on our findings, a rare allele of this variant, significantly associated with lower *ABCA13* intratumoral transcript levels in a validation set (Table 6). Lower transcript levels of *ABCA13* were associated with worse response to NACT in a previous study [14], further underpinning the role of this SNP as a putative poor prognosis biomarker. This consequence is the most interesting link observed at present. Nevertheless, the response to NACT does not significantly associate with DFS in our datasets a fact that clearly calls for further research.

Another variant in *ABCA13,* the missense rs74859514 (Ala2223Pro), associated with DFS in patients treated with chemotherapy, but without gene dosage relationship. Neither of these two SNPs has records in the present literature, although associations of *ABCA13* with patients’ outcome have been described in several previous studies. A decreased expression of *ABCA13* was associated with shorter DFS in 51 glioblastoma patients [19] and 51 colorectal cancer patients [20]. The opposite was found for ovarian cancer (*n* = 77) and higher levels of *ABCA13* predicted worse overall survival in ovarian cancer patients [21]. Amplification of 7p12 (which includes *ABCA13* and *HUS1, EGFR,* and *IKZF1*) predicted worse response to NACT in muscle-invasive bladder cancer [22]. Such contradictory results from different cancers are puzzling. Despite we must bear in mind that none of the associations found in our study passed the FDR test, some may still have clinical potential. Additional studies will be needed to confirm these results.

A synonymous variant rs71428357 in *ABCA12* associated with response to NACT. Patients responding well to chemotherapy were more often carriers of the rare allele. Synonymous variants can affect RNA splicing, folding, and stability [23] and are associated with several diseases, such as Alzheimer disease, pulmonary sarcoidosis, galactosemia, or cancer [24]. The role of this particular *ABCA12* variant in cancer or other diseases is still unknown. However, higher *ABCA12* transcript levels in non-tumor tissues associated with worse response to NACT in breast cancer patients in our previous study [14]. The opposite, i.e., higher levels associating with residual disease, was found by Park et al. [25]. Interestingly, we previously identified this gene among candidate ABCs with predictive or prognostic potential for patients with breast, colorectal, and pancreatic carcinomas [26].

Among other members of the ABCA family, associations with response to NACT were observed for *ABCA4* (variant rs2275032) and *ABCA9* (rs11871944). A deletion in *ABCA7* (rs9282562) associated with shorter DFS of the patients. These variants are not described in the present literature, however, higher transcript levels of *ABCA9* significantly associated with worse survival in high-grade serous ovarian cancer tumors [6]. Silencing of *ABCA7* reduces epithelial to mesenchymal transition in ovarian cancer cell lines and knockdown of *ABCA7* inhibited migration, cell proliferation, and invasion [27]. In addition, lower *ABCA7* levels associated with shorter DFS of colorectal cancer patients [20].

*ABCB5* confers 5-fluorouracil resistance and promotes cell invasiveness in colorectal cancer [28]. Variant rs3210441 in *ABCB5* associated with response to NACT in our study, but no eQTL was found and additional supportive data about the role of this SNP or protein in breast cancer is lacking.

Protein coded by *ABCC11* is responsible for transport of bile acids, conjugated steroids, or cyclic nucleotides. Diseases linked with this gene include malfunction of apocrine gland secretion and lateral sinus thrombosis (https://www.genecards.org). *ABCC11* is a transporter of 5-fluorouracil [3]. In our study, a missense *ABCC11* variant rs17822931 (Gly180Arg) associated with response to NACT. Carriers of the wild-type allele had significantly poorer outcomes than patients with an alternative allele. This variant is known for its determination of human earwax type [29]. It is associated with breast cancer risk in the Japanese population [30]. This variant is also linked with axillary osmidrosis, colostrum secretion in the mammary gland, and mastopathies [31]. Wild type allele C also confers to chemotherapy resistance to 5-fluorouracil by exporting active metabolite 5-fluoro-2′-deoxyuridine 5′-monophosphate (FdUMP) [32]. *ABCC11* expression (together with *ABCB1*) is responsible for resistant phenotype of breast cancer cell lines resistant to eribulin and inhibition of *ABCC11* can partially restore the cross-resistance to 5-fluorouracil [33]. Higher *ABCC11* gene expression was also associated with poor response to NACT in breast cancer patients [25]. Interestingly, this SNP is associated with expression of *ABCC11* only in the brain, but with *LONP2,* coding mitochondrial matrix protein, in breast tissue (Table 7). Relations between mastopathy, breast cancer risk, and, after chemotherapy, even drug resistance suggest strong connection of this variant to breast cancer. Association with response to chemotherapy of breast cancer patients has been suggested previously [31], our result corroborates this assertion.

Among other members of the ABCC family, *ABCC5* (rs4148579) and *ABCC8* (rs739689) associated with response to NACT and *ABCC4* (rs899494) with DFS of the patients. *ABCC4* was among amplified genes in resistant cancer cell lines [34]. The *ABCC4* gene was also identified to play a role in cellular migration of breast cancer cell line models MCF-7 and MDA-MB-231 [35]. In our previous study [14], we have seen associations of high *ABCC8* transcript levels with low grade and negative/positive status of estrogen receptor. Additionally, the expression level non-significantly (*p* = 0.096) associated with worse responses of breast cancer patients to NACT [14]. Nevertheless, in the present study we did not find association of rs739689 (intronic A > G transition) with *ABCC8* transcript levels. eQTL associations at the GTEx portal are ambiguous. The wild-type AA genotype has the highest expression of *ABCC8* in cerebellum, but no significant association can be found in breast tissue. This SNP is also highly significantly associated with expression of *NCR3LG1*, *KCNJ11*, and *SNORD14* genes with fragmentary and elusive data on association with breast cancer. From the data discussed above, it can be summarized that the present knowledge is incomplete and, thus, no clear picture can be presented.

Unlike other ABCD transporters, *ABCD4* is not found in peroxisomes, but in lysosomes. It takes part in transport of cobalamin (vitamin B12) and mutations in this transporter cause inherited defects of intracellular cobalamin metabolism [10]. Low transcript levels of this gene were also associated with shorter DFS of colorectal cancer patients [20] and *ABCD4* was among amplified genes in resistant cancer cell lines [34]. In our study, wild-type variants rs2301347 and rs2301346 (both intronic) associated with the good response to NACT. Wild-type genotypes of these two variants show lower transcript levels of long non-coding (lnc) RNA lnc-SYNDIG1L-2 overlapping *ABCD4* in mammary tissue (Table 7) suggesting potential clinical relevance. However, the lack of association with *ABCD4* transcript levels that we found in our dataset precludes any strict conclusions.

*ABCG8* is a transporter of sterols from hepatocytes and enterocytes [36]. The rare allele of its SNP rs34198326 was associated with longer DFS of chemotherapy treated patients in our study. Expression of *ABCG8* was downregulated in tumors of breast cancer patients compared to non-neoplastic control tissues [14], but the role of germline polymorphism is unclear.

The role of ABC transporters in cancer has been known for a long time. Multidrug resistance has been studied since 1970, when it was first mentioned [37]. The well-studied *ABCB1* gene (MDR1) was discovered in 1974 by V. Ling, and nearly twenty years later, the discovery of *ABCC1* and *ABCG2* concerning drug resistance was reported [2]. Although associations of *ABCB1* gene expression with breast cancer prognosis were reported repeatedly, evidence for the role of its genetic variability in response to treatment is elusive. A recent review demonstrated that three frequently studied polymorphisms in *ABCB1* (rs1045642, rs1128503, and rs2032582) cannot be considered reliable predictors of response to chemotherapy in breast cancer patients [38]. Similarly, an association of *ABCC1* expression with the survival of breast cancer patients was described [39]. However, only a few studies on genetic polymorphisms can be found. *ABCC1* variants rs4148350, rs45511401, and rs246221 associated with the risk of febrile neutropenia in patients treated with 5-fluorouracil, epirubicin, and cyclophosphamide [40] and it was very recently discovered that *ABCC1* variant burden is a strong predictor of DFS in breast cancer patients rather than the genotype attributed to individual variants [41]. *ABCG2* transports several drugs used for breast cancer treatment. In a recent study on the TCGA cohort, *ABCG2* transcript levels associated with a decreased progression-free survival, although, gene variants (either somatic or germline) influenced *ABCG2* expression only moderately [42]. From the above-reviewed information, it can be summarized that despite numerous studies on drug transporters utilization for predicting therapy outcome, strong support is still missing. Other transporters, with rather physiological roles, are much less explored in oncology, and studies were largely dedicated to gene expression in contrast with less studied genetic variability.

In conclusion, genetic variability in ABC transporters might play a role in breast cancer prognosis and help with prediction of therapy outcome of the patients. Although no alterations observed by this study can be considered statistically validated, particularly associations of downstream variant affecting expression, rs17548783 in *ABCA13* with DFS and variant rs17822931 (Gly180Arg) in *ABCC11* with response to NACT attract attention because of their support in the literature. These are interesting candidates for future research. Furthermore, elucidations are needed to explore additional genetic component, e.g., non-coding sequences, copy numbers and structural variations, somatic mutations, etc. of the ABC transporter superfamily.

## 4. Materials and Methods

### 4.1. Patients

The evaluation phase of the study included 105 breast cancer patients, diagnosed in the Institute for the Care for Mother and Child and Medicon, both in Prague and in the Hospital Atlas in Zlin (Czech Republic) over the period of 2006–2013. Of these, 68 patients underwent preoperative (neoadjuvant) treatment with regimens containing 5-fluorouracil, anthracyclines, cyclophosphamide (FAC or FEC), and/or taxanes. The rest received adjuvant postoperative treatment with regimens based on the same drugs. Clinical data of these patients are presented in Table S1.

For the validation phase, we used 802 breast cancer patients, recruited over the period of 2001–2013 from the Institute for the Care for Mother and Child, Medicon, the Motol University Hospital, all in Prague and in the Hospital Atlas in Zlin (all in the Czech Republic). Patients received either neoadjuvant or adjuvant chemotherapy or by hormonal therapy. Clinical data of these patients are presented in Table S3.

More details about the patient recruitment can be found elsewhere [13]. DFS was defined as the time between surgery and first disease relapse including local relapses. Response to NACT was evaluated by the Response Evaluation Criteria in Solid Tumors (RECIST [43]) based on imaging data retrieved from medical records.

Procedures performed in the present study were in accordance with the 1964 Helsinki declaration and its later amendments or comparable ethical standards. Study protocol was approved by the Ethical Commission of the National Institute of Public Health in Prague (approvals no. 9799-4, 15-25618A, and 17-28470A). All patients were informed about the study and those who agreed and signed an informed consent further participated in the study.

### 4.2. Panel Sequencing—Evaluation Phase

Blood samples were collected during the diagnostic procedures using tubes with K3EDTA anticoagulant and genomic DNA was isolated from human peripheral blood lymphocytes by the standard phenol/chloroform extraction and ethanol precipitation.

In the evaluation phase, raw data for 48 ABC transporter genes and one pseudogene were extracted from the previously published study [13]. Briefly, reads were mapped on reference sequence hg19 using Burrows-Wheeler Alignment (BWA) mem [44], base and indel recalibration and short indels and SNVs discovery was done in the Genome Analysis Toolkit (GATK) [45] and annotation of variants was done using Annovar [46] (for details of the library preparation, target enrichment, data processing, and variant calling, see [13]).

### 4.3. Genotyping—Validation Phase

In total, 42 genetic variants were selected for the validation phase and assessed using commercially provided competitive allele specific PCR (KASP™) genotyping (LGC Genomics, Hoddesdon, UK) in DNA samples from 802 breast cancer patients. Primers and probes were designed by the service provider. 10% of the samples were analyzed in duplicates for the purpose of the quality control. The genotyping concordance between duplicate samples exceeded 99%.

### 4.4. Statistical Analyses

In the evaluation phase, DFS was calculated with respect to the groups of patients divided by the genotype (common homozygous, heterozygous, and rare homozygous). The log-rank test for each variant was performed and the Kaplan–Meier plot was generated for visual inspection of gene dosage. We set the study follow-up end to 120 months (10 years) and thus, all subjects with DFS exceeding 120 months were censored. The response of patients to NACT was set to “good” in the case of complete or partial pathological remission (CR/PR) and “poor” for stable or progressive disease (SD/PD). We evaluated associations between genotypes (common homozygous, heterozygous, and rare homozygous) and response using the Pearson chi-square test. Adjusted *p*-value was calculated for each variant and each of these tests. Adjusted *p*-value for the log-rank test was based on 100 permutations of original data. A *p*-value of less than 0.05 after adjustment for multiple testing was considered statistically significant. Variants significantly associating with either DFS or response to NACT in the evaluation phase entered the validation phase of the study.

In the validation phase, the Pearson chi-square test and the log-rank tests were used as described above. For the evaluation of allele effect, recessive, dominant, co-dominant, and additive genetic models were used. Association of variants with transcript levels was assessed by the non-parametric Kruskal–Wallis test. Adjusted *p*-values were calculated using Benjamini–Hochberg false discovery rate (the FDR test) as a correction for multiple testing [47]. Haplotype analysis was conducted in HaploView 4.2 (Broad Institute, Cambridge, MA, USA). Statistical analyses were conducted using R and the statistical program SPSS v16.0 (SPSS, Chicago, IL, USA).

The sequencing data that support the findings of this study are openly available in Sequence Read Archive (SRA, https://www.ncbi.nlm.nih.gov/sra) under accession no. PRJNA510917.

## Figures and Tables

**Table 1:** Observed alterations in ATP-binding cassette (ABC) transporters divided by function according to Annovar.

Type	Total	Percentage
Intronic	1544	59.1
Exonic (coding)	636	24.4
UTR’3	204	7.8
Intergenic	76	2.9
UTR’5	49	1.9
Downstream ^1^	28	1.1
Upstream ^1^	26	1.0
Splicing ^2^	13	0.5
Other ^3^	35	1.3

^1^ Variants are 1 kb from transcription end/start site; ^2^ Variants are 2 bp within splice junction. ^3^ Exonic/intronic non-coding RNA, or variant in overlapping regions (upstream–downstream) of two different genes.

**Table 2:** Overview of the observed exonic alterations in ABC transporters by coding consequence.

Classification	Total	Percentage
Non-synonymous SNV	355	55.8
Synonymous SNV	263	41.4
Stop-gain	8	1.3
Frameshift deletion	6	0.9
Frameshift insertion	3	0.5
Non-frameshift deletion	1	0.1

**Table 3:** Distribution of genotypes for variants assessed in the validation phase.

Gene	SNP ID ^1^		Genotype Distribution ^2^		MAF ^3^	
		Common Homozygotes	Heterozygotes	Rare Homozygotes	Validation Set	Testing Set
*ABCA1*	rs41474449	658	136	3	0.09	0.07
*ABCA4*	rs537831	377	342	78	0.31	0.31
*ABCA4*	rs2065711	436	309	55	0.26	0.20
*ABCA4*	rs2275032	540	230	30	0.18	0.14
*ABCA4*	rs2275033	270	396	133	0.41	0.40
*ABCA4*	rs3789398	353	361	84	0.33	0.35
*ABCA5*	rs1420904	679	113	7	0.08	0.08
*ABCA5*	rs2067851	681	112	2	0.07	0.07
*ABCA7*	rs9282562	604	188	8	0.13	0.14
*ABCA8*	rs4147976	318	358	121	0.38	0.35
*ABCA9*	rs2302294	368	352	80	0.32	0.34
*ABCA9*	rs11871944	326	366	103	0.36	0.40
*ABCA12*	rs71428357	726	70	3	0.05	0.08
*ABCA13*	rs7780299	597	187	16	0.14	0.12
*ABCA13*	rs17132289	687	106	6	0.07	0.08
*ABCA13*	rs17548783	201	400	192	0.49	0.49
*ABCA13*	rs28637820	628	163	8	0.11	0.13
*ABCA13*	rs74859514	665	124	10	0.09	0.08
*ABCB1*	rs9282564	609	168	21	0.13	0.13
*ABCB5*	rs3210441	283	400	116	0.40	0.44
*ABCB5*	rs12700230	466	285	49	0.24	0.23
*ABCB5*	rs2893007	676	120	5	0.08	0.10
*ABCB8*	rs2303922	336	362	100	0.35	0.34
*ABCB11*	rs853772	203	403	190	0.49	0.25
*ABCC1*	rs4148379	456	287	48	0.24	0.20
*ABCC2*	rs2273697	478	273	39	0.22	0.22
*ABCC3*	rs8077268	649	147	4	0.10	0.10
*ABCC3*	rs12604031	271	374	154	0.43	0.44
*ABCC4*	rs899494	583	198	17	0.15	0.12
*ABCC4*	rs2274405	339	352	102	0.35	0.37
*ABCC5*	rs4148579	259	404	137	0.42	0.43
*ABCC5*	rs12638017	686	111	3	0.07	0.06
*ABCC8*	rs739689	349	356	91	0.34	0.40
*ABCC10*	rs75320251	654	135	8	0.09	0.09
*ABCC11*	rs17822931	592	184	21	0.14	0.14
*ABCC13*	rs2254297	254	381	160	0.44	0.40
*ABCC13*	rs2822582	306	369	121	0.38	0.40
*ABCD4*	rs2301346	394	334	67	0.29	0.32
*ABCD4*	rs2301347	305	376	120	0.38	0.40
*ABCF2*	rs79537035	527	242	30	0.19	0.23
*ABCG8*	rs34198326	685	109	4	0.07	0.06
*ABCG8*	rs56260466	685	104	9	0.08	0.06
*CFTR*	rs34855237	538	229	30	0.18	0.08

^1^ Reference number in dbSNP (https://www.ncbi.nlm.nih.gov/snp/); ^2^ Genotypes do not sum up to 802 due to missing data; ^3^ MAF = minor allele frequency.

**Table 4:** Validated variants in ABC transporters significantly associating with the response of patients to the neoadjuvant cytotoxic therapy.

Gene	SNP ID	Genotype	Good Response ^1^	Poor Response ^1^	χ-Square	*p*-Value
*ABCA4*	rs2275032	AA	75	33	6.33	0.042
		CA	48	7		
		CC	4	1		
*ABCA9*	rs11871944	CC	48	24	6.76	0.034
		CT	61	14		
		TT	18	2		
*ABCA12*	rs71428357	CC	103	41	8.32	0.004
		CT + TT	22	0		
*ABCB5*	rs3210441	GG	42	11	6.22	0.045
		GA	62	28		
		AA	23	2		
*ABCC5*	rs4148579	CC	43	6	8.55	0.014
		CT	68	24		
		TT	15	11		
*ABCC8*	rs739689	AA	55	11	6.81	0.033
		GA	60	21		
		GG	11	9		
*ABCC11*	rs17822931	CC	89	37	6.42	0.011
		CT + TT	37	4		
*ABCD4*	rs2301347	CC	53	9	8.59	0.014
		CA	60	21		
		AA	14	11		
*ABCD4*	rs2301346	TT	73	15	7.28	0.026
		CT	45	20		
		CC	7	6		

^1^ Numbers of patients with specified genotypes divided by response to the neoadjuvant treatment.

**Table 5:** Validated associations of variants in ABC transporters associating with DFS of patients treated with cytotoxic therapy according to their molecular subtypes.

Gene	SNP ID	Genotypes	Subtypes ^1^			
			Luminal A	Luminal B	HER2	TNBC
All patients (*n* = 744)						
*ABCA7*	rs9282562	Common homozygous	165	206	39	63
		Rare allele	45	68	17	21
		*p*-value ^2^	0.626	0.316	**0.010**	0.325
*ABCA13*	rs17548783	Common homozygous	58	67	12	24
		Heterozygous	112	129	31	40
		Rare homozygous	39	74	13	20
		*p*-value ^2^	0.050	0.114	0.492	**0.039**
Patients treated with cytotoxic therapy (*n* = 371)						
*ABCA13*	rs74859514	Common homozygous	62	125	25	58
		Rare allele	12	27	11	8
		*p*-value ^2^	0.441	0.606	**0.001**	**0.009**
*ABCC4*	rs899494	Common homozygous	50	114	27	46
		Rare allele	24	38	9	20
		*p*-value ^2^	0.825	0.415	0.050	0.565
*ABCG8*	rs34198326	Common homozygous	63	135	30	54
		Rare allele	11	17	5	11
		*p*-value ^2^	0.094	**0.040**	0.847	0.091

^1^ Numbers of patients for each genotype stratified by molecular subtypes are displayed; HER2 = HER2 enriched, TNBC = triple negative breast cancer. ^2^ *p*-value departed from log-rank test (significant results in bold).

**Table 6:** Association of single nucleotide polymorphism (SNP) rs17548783 in ABCA13 transporter with intratumoral transcript levels.

SNP ID	Genotype	*n*	Expression ^1^	S.D. ^2^	*p*-Value
rs17548783	Common homozygous	9	−7.29	2.11	0.015
	Heterozygous	18	−9.90	2.67	-
	Rare homozygous	7	−9.45	2.67	-

^1^ Transcript levels expressed as Ct (cycle threshold) value of ABCA13 subtracted from mean Ct of reference genes [14]. ^2^ S.D. = Standard deviation.

**Table 7:** eQTL analysis of SNPs significantly associating with patients’ DFS or response to neoadjuvant cytotoxic therapy (NACT).

SNP ID	Gene	Tissue	Normalized Expression Trend	*p*-Value ^1^
rs11871944	*ABCA9*	multiple ^2^	CC > TC > TT	3.1 × 10^−7^
rs4148579	*ABCC5*	multiple ^2^	CC > TC > TT^3^	3.5 × 10^−33^
rs739689	*ABCC8*	brain (cerebellum)	AA > AG > GG	8.6 × 10^−9^
rs17822931	*ABCC11*	brain	CC > CT > TT	6.9 × 10^−5^
	*LONP2 ^4^*	breast	CC > CT > TT	1.4 × 10 ^−4^
rs2301347	*ABCD4*	multiple ^2^	AA > CA > CC	5.0 × 10^−20^
	*lnc-SYNDIG1L-2 ^4^*	breast	AA > CA > CC	1.8 × 10^−16^
rs2301346	*ABCD4*	multiple ^2^	CC > TC > TT	1.8 × 10^−12^
	*lnc-SYNDIG1L-2 ^4^*	breast	CC > TC > TT	4.2 × 10^−15^
rs9282562	*ABCA7*	multiple ^2^	ref > het > delTG	1.3 × 10^−11^
s74859514	*UPP1 ^4^*	cerebellum, muscle	GG > GC > CC	2.7 × 10^−5^
rs899494	*ABCC4*	thyroid, whole blood	AA > AG > GG	1.9 × 10^−16^

^1^*p*-value of the most significant association is shown. ^2^ Significant results in more than three different tissues. ^3^ The highest expression is seen for TT genotype in whole blood and esophageal mucosa; the opposite i.e., highest expression of CC genotype is seen for the rest of the tissues. ^4^ Alternative eQTL.

## Supplementary Materials

Supplementary Materials can be found at https://www.mdpi.com/1422-0067/21/24/9556/s1.

## Abbreviations

ABC	ATP-binding cassette
DFS	Disease-free survival
FDR	false discovery rate
LOF	Loss-of-function
MAF	minor allele frequency
NGS	next generation sequencing
SNP	single nucleotide polymorphism
SNV	single nucleotide variant
UTR	untranslated regions
