# Supplementary material for: Role of Genetic Variation in ABC Transporters in Breast Cancer Prognosis and Therapy Response
Source: Int J Mol Sci. 2020 Dec 15;21(24):9556. doi: 10.3390/ijms21249556 (PMC7765380; doi:10.3390/ijms21249556)

International Journal of *Molecular Sciences*

MDPI

Supplementary material

Role of Genetic Variation in ABC Transporters in Breast Cancer Prognosis and Therapy Response

Viktor Hlaváč [1,2], Radka Václavíková [1,2], Veronika Brynychová [1,2], Renata Koževnikovová [3], Katerina Kopečková [4], David Vrána [5], Jiří Gatěk [6] and Pavel Souček [1,2,*]

[1] Toxicogenomics Unit, National Institute of Public Health, Prague, Czech Republic; viktor.hlavac@szu.cz (V.H.); rvaclavikova@seznam.cz (R.V.); veronikabrynychova@seznam.cz (V.B.)

[2] Biomedical Center, Faculty of Medicine in Pilsen, Charles University, Pilsen, Czech Republic

[3] Department of Oncosurgery, MEDICON, Prague, Czech Republic; renata.kozevnikovova@onko-centrum.cz (R.K.)

[4] Department of Oncology, Second Faculty of Medicine, Charles University and Motol University Hospital, Prague, Czech Republic; katerina.kopeckova@fnmotol.cz (K.K.)

[5] Department of Oncology, Palacky University Medical School and Teaching Hospital, Olomouc, Czech Republic; davvrana@gmail.com (D.V.)

[6] Department of Surgery, EUC Hospital and University of Tomas Bata in Zlin, Zlin, Czech Republic; gatekj@gmail.com (J.G.)

* Correspondence: pavel.soucek@szu.cz; Tel.: +420-267-082-711, ORCID: 0000-0002-4294-6799

Received: date; Accepted: date; Published: date

International Journal of Molecular Sciences

Supplementary Table S1. Clinical data of patient in the testing set.

Characteristics	Patients, N (%)[1]
Age at diagnosis, mean ± S.D.[2] (years)	51.7 ± 9.4
Menopausal status	
Premenopausal	46 (46)
Postmenopausal	55 (55)
Missing data	4
Tumor size (pT)	
pTis	8 (8)
pT1	50 (48)
pT2	40 (39)
pT3	5 (5)
pTX	2
Lymph node metastasis (pN)	
Absent (pN0)	68 (65)
Present (pN1-3)	37 (35)
Pathological stage	
SI	46 (44)
SII	47 (45)
SIII	12 (11)
Histological type	
Invasive ductal carcinoma	88 (84)
Other type	17 (16)[4]
Pathological grade	
G1	11 (11)
G2	35 (35)
G3	54 (54)
GX	5
Estrogen receptor status	
Positive	38 (38)
Negative	61 (62)
Missing data	6
Progesterone receptor status	
Positive	39 (39)
Negative	60 (61)
Missing data	6
Expression of HER2	
Positive	2 (2)
Negative	97 (97)
Missing data	6
Expression of Ki-67, mean ± S.D.[2] (%)	32.9 ± 20.3

International Journal of
Molecular Sciences

Supplementary Table S1. Clinical data of patient in the testing set. (Cont.).

Characteristics	Patients, N (%)[1]
Molecular subtype	
Luminal A	11 (11)
Luminal B	30 (30)
Triple negative	58 (59)
Missing data	6
Response to neoadjuvant cytotoxic therapy	
Complete or partial response	47 (69)
Stable disease or progression	21 (31)
Not applicable[3]	37

Footnotes:
[1] Number of patients with % in parentheses
[2] S.D.=standard deviation
[3] Patients treated with adjuvant therapy without neoadjuvant cytotoxic therapy
[4] Six lobular, six medullary, two metaplastic, one mucinous, one papillary, and one neuroendocrine invasive carcinomas

International Journal of
Molecular Sciences

MDPI

Supplementary Table S2. Prioritized variants for the validation phase.

Gene	HGVS coding (GRCh38)	HGVS protein	Classification[1]	Rs ID[2]	Response[3]	DFS[3]	MAF[4]	GnomAD[5]
ABCA1	NC_000009.12:g.104781418_104781420del	—	3'UTR	rs41474449	NS	0.049	0.07	0.08
ABCA4	NC_000001.11:g.94011139C>T	—	intron	rs2065711	0.010	NS	0.20	0.25
ABCA4	NC_000001.11:g.94008629A>C	—	intron	rs2275032	0.008	NS	0.14	0.18
ABCA4	NC_000001.11:g.94014481C>T	—	intron	rs2275033	NS	0.040	0.40	0.43
ABCA4	NC_000001.11:g.94045977G>A	—	intron	rs3789398	0.015	NS	0.35	0.33
ABCA4	NC_000001.11:g.94010999G>A	—	intron	rs537831	0.018	NS	0.31	0.29
ABCA5	NC_000017.11:g.69249800G>A	—	intron	rs1420904	0.047	NS	0.08	0.10
ABCA5	NC_000017.11:g.69249759T>C	—	intron	rs2067851	NS	0.020	0.07	0.09
ABCA7	NC_000019.10:g.1051139_1051140TG[1]	—	intron	rs9282562	NS	0.040	0.14	0.11
ABCA8	NC_000017.11:g.68929782C>T	—	intron	rs4147976	NS	0.049	0.35	0.42
ABCA9	NC_000017.11:g.69061147T>C	—	intron	rs11871944	0.021	NS	0.40	0.37
ABCA9	NC_000017.11:g.68989851T>G	**p.Lys1306Thr**	**missense**	rs2302294	NS	0.030	0.34	0.35
ABCA12	NC_000002.12:g.214989432C>T	—	synonymous	rs71428357	0.014	NS	0.08	0.05
ABCA13	NC_000007.14:g.48389118A>T	**p.Tyr3851Phe**	**missense**	rs17132289	NS	0.030	0.08	0.07
ABCA13	NC_000007.14:g.48410560T>C	—	synonymous	rs17548783	NS	0.040	0.49	0.48
ABCA13	NC_000007.14:g.48198124G>C	—	intron	rs28637820	0.029	NS	0.13	0.13
ABCA13	NC_000007.14:g.48276333G>C	**p.Ala2223Pro**	**missense**	rs74859514	NS	<0.001	0.08	0.09
ABCA13	NC_000007.14:g.48392165C>T	—	intron	rs7780299	NS	0.010	0.12	0.14
ABCB1	NC_000007.14:g.87600124T>C	**p.Asn21Asp**	**missense**	rs9282564	NS	0.030	0.13	0.11
ABCB5	NC_000007.14:g.20700049G>A	—	intron	rs12700230	0.008	NS	0.23	0.23
ABCB5	NC_000007.14:g.20661065A>T	—	intron	rs2893007 [6]	NS	0.03 [6]	0.10	0.08
ABCB5	NC_000007.14:g.20756864G>A	—	3'UTR	rs3210441	0.037	NS	0.44	0.36
ABCB8	NC_000007.14:g.151028635A>C	—	intron	rs2303922	NS	0.049	0.34	0.36
ABCB11	NC_000002.12:g.168958145G>A	—	intron	rs853772	0.034	NS	0.25	0.48
ABCC1	NC_000016.10:g.16138351del	—	intron	rs4148379	NS	0.049	0.20	0.22
ABCC2	NC_000010.11:g.99804058G>A	**p.Val417Ile**	**missense**	rs2273697	0.031	NS	0.22	0.20

International Journal of
Molecular Sciences

MDPI

Supplementary Table S2. Prioritized variants for the validation phase. (Cont.)

Gene	HGVS coding (GRCh38)	HGVS protein	Classification[1]	Rs ID[2]	Response[3]	DFS[3]	MAF[4]	GnomAD[5]
ABCC3	NC_000017.11:g.50635344G>A	—	intron	rs12604031	0.034	NS	0.44	0.40
ABCC3	NC_000017.11:g.50676161C>T	—	intron	rs8077268	NS	0.020	0.10	0.11
ABCC4	NC_000013.11:g.95206724T>C	—	synonymous	rs2274405	NS	0.030	0.37	0.34
ABCC4	NC_000013.11:g.95209550A>G	—	synonymous	rs899494	NS	0.030	0.12	0.14
ABCC5	NC_000003.12:g.184000591A>G	—	intron	rs12638017	NS	0.010	0.06	0.07
ABCC5	NC_000003.12:g.183967461T>C	—	intron	rs4148579	0.044	NS	0.43	0.46
ABCC8	NC_000011.10:g.17395957A>G	—	intron	rs739689	0.041	NS	0.40	0.35
ABCC10	NC_000006.12:g.43427363G>A	—	upstream	rs75320251	0.045	NS	0.09	0.11
ABCC11	NC_000016.10:g.48224287C>T	**p.Gly180Arg**	**missense**	rs17822931	0.047	NS	0.14	0.13
ABCC13	NC_000021.9:g.14279748A>G	—	intron	rs2254297	NS	0.049	0.40	0.43
ABCC13	NC_000021.9:g.14337293T>G	—	intron	rs2822582	NS	0.020	0.40	0.37
ABCD4	NC_000014.9:g.74299377T>C	—	intron	rs2301346	0.012	NS	0.32	0.30
ABCD4	NC_000014.9:g.74299190C>A	—	intron	rs2301347	0.003	NS	0.40	0.37
ABCF2	NC_000007.14:g.151218016C>T	—	intron	rs79537035	0.032	NS	0.23	0.17
ABCG8	NC_000002.12:g.43874090A>G	—	intron	rs34198326	NS	0.040	0.06	0.07
ABCG8	NC_000002.12:g.43852262G>A	—	intron	rs56260466	NS	0.049	0.06	0.06
CFTR	NC_000007.14:g.117559403A>G	—	intron	rs34855237	NS	0.03	0.08	0.21

Footnotes:

1 Classification in Annovar

2 SNV number in dbSNP (https://www.ncbi.nlm.nih.gov/snp/)

3 p-value provided for clinical associations; NS = non-significant

4 MAF = minor allele frequency in the testing set

5 The Genome Aggregation Database (gnomAD), allelic frequencies in European non-Finnish population

6 Variant replacing failed rs11764054 based on tagging analysis; rs11764054 associated with DFS (p = 0.030)

International Journal of
Molecular Sciences

MDPI

Supplementary Table S3. Clinical data of patients in the validation set.

Characteristics	Patients, N (%)[1]
Age at diagnosis, mean ± S.D.[2] *(years)*	58.9 ± 12.5
Menopausal status	
Premenopausal	196 (25)
Postmenopausal	592 (75)
Missing data	14
Tumor size (pT)	
pTis	65 (8)
pT1	488 (62)
pT2	206 (27)
pT3	18 (2)
pT4	10 (1)
pTX	15
Lymph node metastasis (pN)	
Absent (pN0)	507 (67)
Present (pN1-3)	252 (33)
pNX	43
Pathological stage	
S0	61 (8)
SI	343 (46)
SII	282 (38)
SIII	64 (9)
SIV	1 (0)
Not determined	51
Histological type	
Invasive ductal carcinoma	596 (75)
Other type	196 (25)
Missing data	10
Pathological grade	
G1	177 (23)
G2	382 (50)
G3	209 (27)
GX	34
Estrogen receptor status	
Positive	615 (77)
Negative	181 (23)
Missing data	6
Progesterone receptor status	
Positive	577 (73)
Negative	219 (27)
Missing data	6

International Journal of
Molecular Sciences

MDPI

Supplementary Table S3. Clinical data of patients in the validation set. (Cont.)

Characteristics	Patients, N (%)[1]
Expression of HER2	
Positive	194 (24)
Negative	600 (76)
Missing data	8
Expression of Ki-67, mean ± S.D.[2] (%)	23.3 ± 22.6
Molecular subtype	
Luminal A	320 (40)
Luminal B	315 (40)
Triple negative	94 (12)
HER2	63 (8)
Missing data	10
Response to neoadjuvant cytotoxic therapy	
Complete or partial response	127 (76)
Stable disease or progression	41 (24)
Not applicable[3]	634

Footnotes:

[1] Number of patients with % in parentheses

[2] S.D.=standard deviation

[3] Patients treated with adjuvant therapy without neoadjuvant cytotoxic therapy

International Journal of
Molecular Sciences
MDPI

Figure S1. Kaplan-Meier survival plots showing significant associations of validated variants with DFS of breast patients stratified according to their molecular subtypes.

All patients (n = 744)

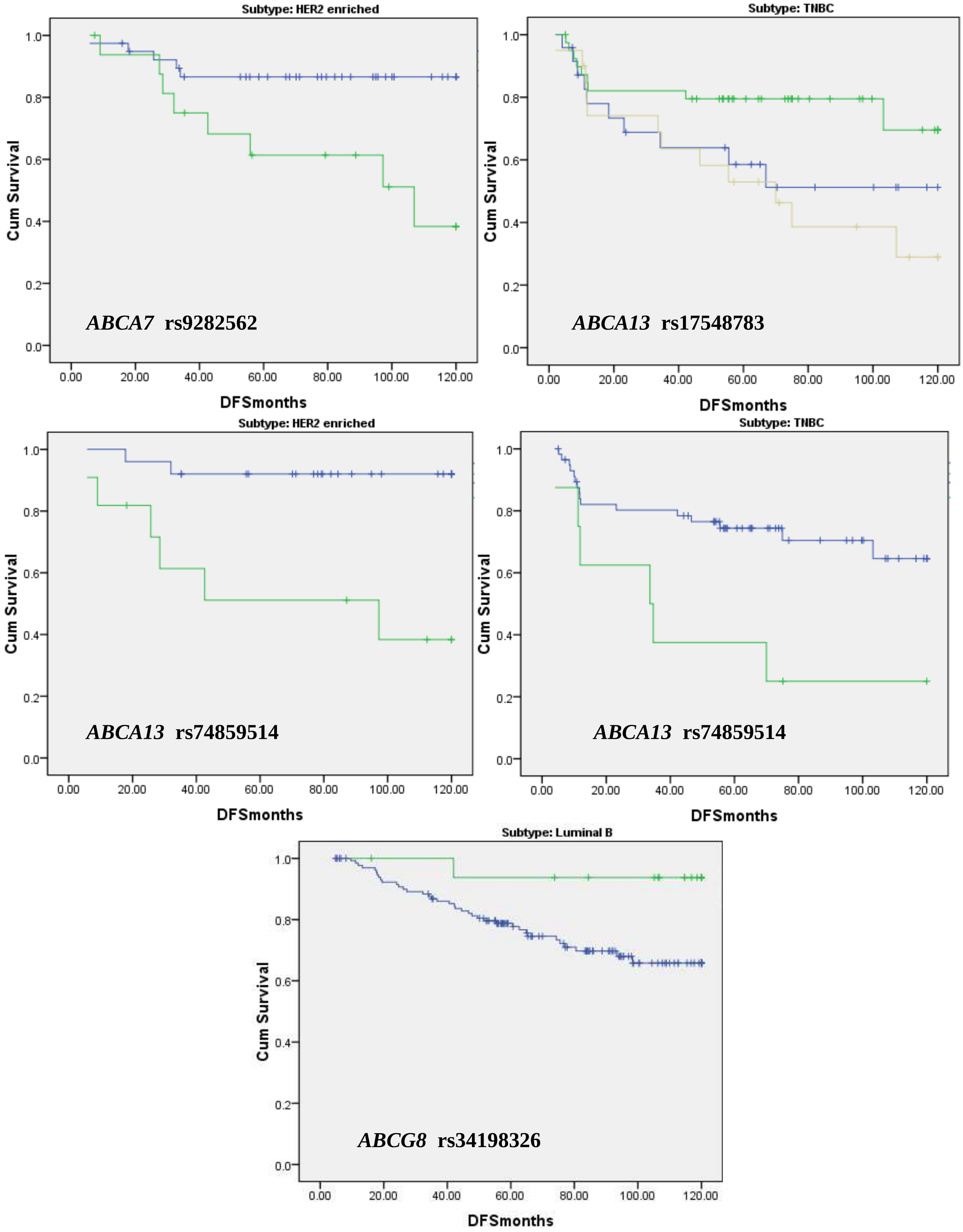

Blue line = common homozygotes; green line = heterozygotes or rare allele carriers; yellow line = rare homozygotes. For all SNPs except *ABCA13* rs17548783 recessive genetic model is displayed.

Figure S2. Significant associations of gene expression in healthy tissues with variants significantly associated with survival or response to cytotoxic chemotherapy – plots are derived from an eQTL analysis at GTEx portal.

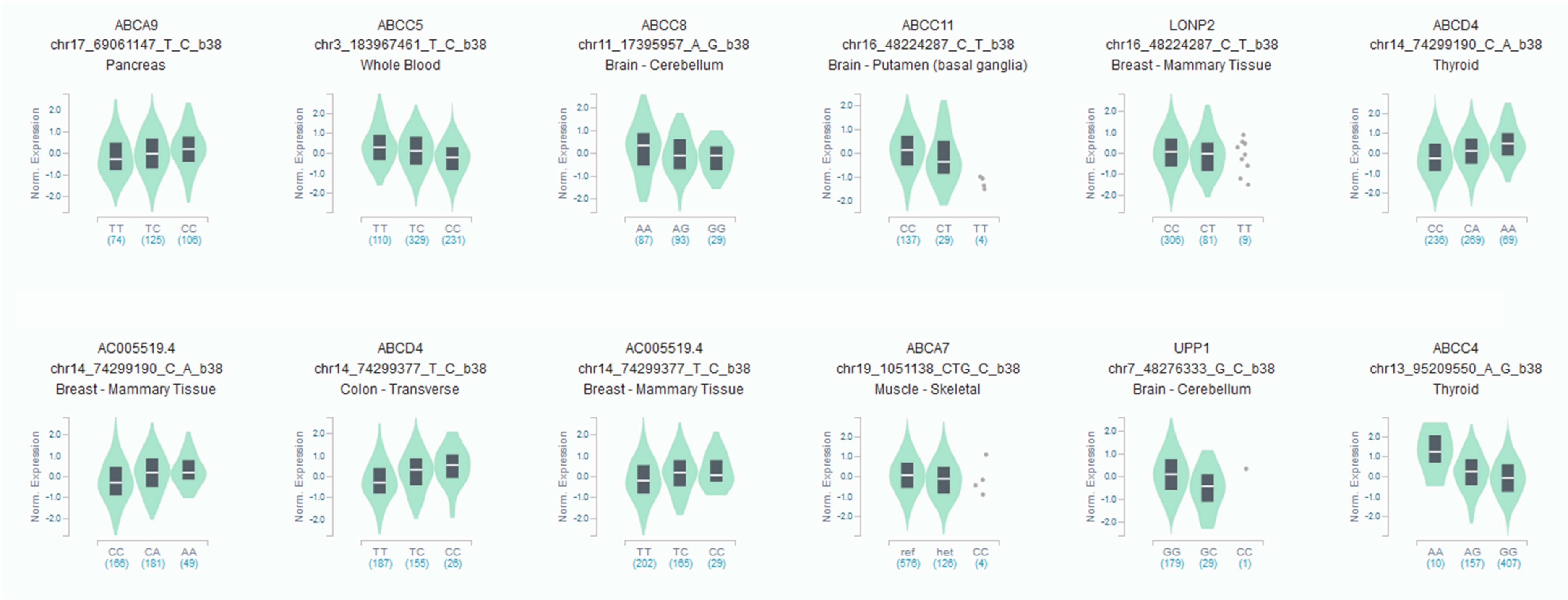

Supplement: Supplementary file 1 [file ijms-21-09556-s001.pdf]
